# The memory effect of magnetoelectric coupling in FeGaB/NiTi/PMN-PT multiferroic heterostructure

**DOI:** 10.1038/srep20450

**Published:** 2016-02-05

**Authors:** Ziyao Zhou, Shishun Zhao, Yuan Gao, Xinjun Wang, Tianxiang Nan, Nian X. Sun, Xi Yang, Ming Liu

**Affiliations:** 1 Electronic Materials Research Laboratory, Key Laboratory of the Ministry of Education & International Center for Dielectric Research, Xi’an Jiaotong University, Xi’an 710049, China; 2 Electrical and Computer Engineering Department, Northeastern University, Boston, MA 02115, USA; 3 Center of Microwave and Millimeter-Wave Technology, School of Information and Electronics, Beijing Institute of Technology, Beijing 100081, China; 4 Collaborative Innovation Center of High-End Manufacturing Equipment, Xi’an Jiaotong University, Xi’an 710049, China

## Abstract

Magnetoelectric coupling effect has provided a power efficient approach in controlling the magnetic properties of ferromagnetic materials. However, one remaining issue of ferromagnetic/ferroelectric magnetoelectric bilayer composite is that the induced effective anisotropy disappears with the removal of the electric field. The introducing of the shape memory alloys may prevent such problem by taking the advantage of its shape memory effect. Additionally, the shape memory alloy can also “store” the magnetoelectric coupling before heat release, which introduces more functionality to the system. In this paper, we study a FeGaB/NiTi/PMN-PT multiferroic heterostructure, which can be operating in different states with electric field and temperature manipulation. Such phenomenon is promising for tunable multiferroic devices with multi-functionalities.

Recently, multiferroic composites with combined ferroelectric (FE) and ferromagnetic (FM) phase have attracted a lot of interests due to strong magnetoelectric (ME) coupling at room temperature^1^,^2^,^3^,^4^,^5^,^6^,^7^. The magnetic properties of the ferromagnetic material can be varied in a large scale by using a non-power-consuming electric field (E-field) through ME effect. Such phenomena is of importance to traditional magnetic tunable devices, as the new concept may somehow lead to the disposal of bulky electromagnets or a magnetic coil pair in those devices, and thus result in a smaller form-factor and power efficient device profile. The ME effect has been widely employed to device applications, and many device prototypes are reported, such as RF/microwave components^8^,^9^,^10^,^11^,^12^,^13^,^14^,^15^, sensors^16^,^17^, magnetoelectric random access memories (MERAMs)^18^,^19^,^20^,^21^,^22^,^23^, etc. For tunable RF/microwave applications, large frequency domain tunability is preferred. Therefore, researches are mainly focused on developing heterostructures with large ME coupling coefficients. Although ferrite/ferroelectric bilayer composites exhibits very low loss, the ME coupling coefficients are mostly limited to several Oe∙cm∙kV^−1^ due to the small magnetostriction value of the oxides^24^. For instance, the tunability of the corresponded devices are up to 200 MHz (10%) for filters^25^, and 180° for phase shifters^26^. To overcome such issue, metal/ferroelectric heterostructures are developed and reported with large ME coupling coefficients^27^,^28^,^29^,^30^,^31^,^32^,^33^,^34^,^35^,^36^,^37^,^38^,^39^,^40^,^41^,^42^,^43^,^44^,^45^,^46^, which has potential for RF/microwave applications. Among those heterostructures, low-loss Iron Gallium Boron (FeGaB)/lead zinc niobate–lead titanate (PZN-PT) bilayer composite has a maximum ME coupling coefficient up to 94 Oe∙cm∙kV^−1^^27^. The ferromagnetic resonant (FMR) spectra of the bilayer may shift from 1.75 GHz to 7.57 GHz under an E-field from 0 to 6 kV/cm. Device prototypes based on FeGaB are reported in our previous work, however, there is no ferroelectric phase involved in the experiment^47^. Another problem in such heterostructure based devices is that when the E-field disappears with the removal of the applied voltage, the ME induced anisotropy does not sustain, and the device’s output, for instance the frequency or the phase shift, goes back to the initial state. Therefore, a certain voltage must be maintained in order to keep the device operating in the expected state, resulting unwanted energy consumption.

Shape memory alloys (SMAs) such as Nickel Titanium (NiTi) may provide a solution in maintaining the device’s functionality after the removal of the voltage^48^,^49^,^50^,^51^,^52^,^53^,^54^,^55^,^56^,^57^. NiTi shape memory materials exhibit various applications, such as manipulation micro-robotics^58^,^59^. These alloys have displacive crystalline phase transformation dominated by shear between a low symmetry product phase and a high symmetry parent phase. Large strains mechanically created in the low temperature can be recovered by one way memory effect through reorientation. This can be finished by heat treatment^60^,^61^. Like most SMAs, different mechanical behavior depending on whether they were tested in austenitic or the martensitic phases is shown in NiTi alloys. NiTi has three different stress/stain curves. An initial low plateau results from the stress induced growth of one martensite orientation. With temperature goes higher, NiTi alloys become the second state, the stress in this region is linear. By changing the temperature (such as applying laser impulses), the strain state of NiTi can be switched back and forth with non-volatility. This unique mechanism raise up a question: can different strain state affect the strain/stress induced ME coupling? Moreover, can ME coupling effect be stored at certain condition and released at another appropriate condition with manipulating strain of SMAs through temperature? The answer is, excitedly, yes.

In this work, we report a FeGaB/NiTi/PMN-PT multiferroic heterostructure with a memorable E-field tuning of magnetic anisotropy. The X-band FMR field up-shifted from 1158 Oe to 1392 Oe with the E-field changing from 0 to 8 kV/cm, and down shifted to 1318 Oe after the E-field is set to zero. This phenomenon implies the ME coupling strength is “locked” by NiTi alloy. Without NiTi, the ME coupling induced FMR field shift is shifted within a magnitude range of ~230 Oe, upto FMR field of 980 Oe; in contrast, with NiTi shape memory alloy, the ME coupling induced FMR shift has a magnitude of ~160 Oe. We define this phenomenon as “memory effect” of NiTi that memorize the ME coupling tunability of multiferroic heterostructure. As the multiferroic structure is heated to 200 °C, the NiTi strain/stress is released and FMR field goes back to ~1160 Oe. By introducing shape memory alloy NiTi, a novel functionality is discovered in multiferroic heterostructure and devices. For instance, the tunability of ME devices that can be switched back and forth at different temperature state corresponding to different NiTi strain state. In FeGaB/NiTi/PMN-PT multiferroic heterostructure, the strain of NiTi can be released at high temperature environment or by applying a temperature (laser impulse), therefore, the “locked” small ME tunability was unlocked to large ME tunability state. Intelligential ME devices like ME-memories and tunable RF/microwave components can be designed, for example, the ME tunable communication devices in satellites/spaceships can be switched with “on” and “off” state at different locations like perihelion or aphelion in the orbit. The ME devices can be deactivated at low temperature (room temperature) and activated through laser impulses or at high temperature environment. Our work reveals a phenomenon of memory effect of SMAs based multiferroic heterostructure that may open a bright future for advanced ME devices with creative functionality.

## Results

**Figure 1:**
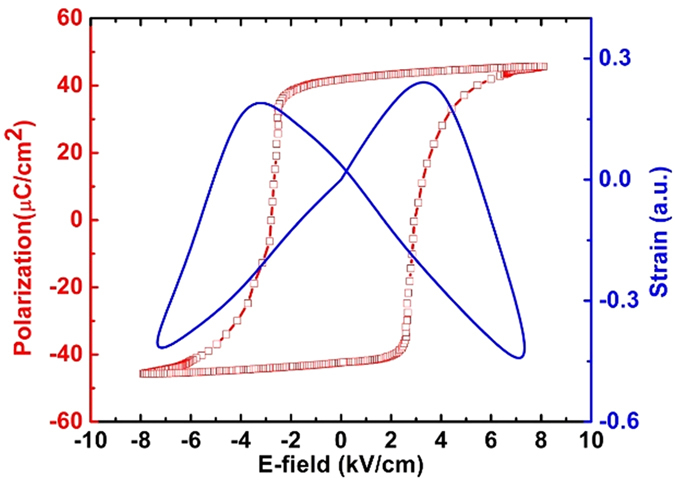
P-E loop and strain loop of PMNPT in this experiment.

The polarization-electric (P-E) and strain vs. E-field curve of the PMNPT ferroelectric substrate is measured, as shown in Fig. 1. A varied voltage from −400 V to 400 V is applied along the thickness direction of the PMN-PT single crystal slab, which corresponds to an E-field from −8 to 8 kV/cm. The P-E loop shows a remnant polarization of ~40 μC/cm^2^ and a coercive E-field (E_c_) of 2.5 kV/cm. The butterfly-like in-plane stain curve is also measured showing a complete ferroelectric domain switching process.

**Figure 2:**
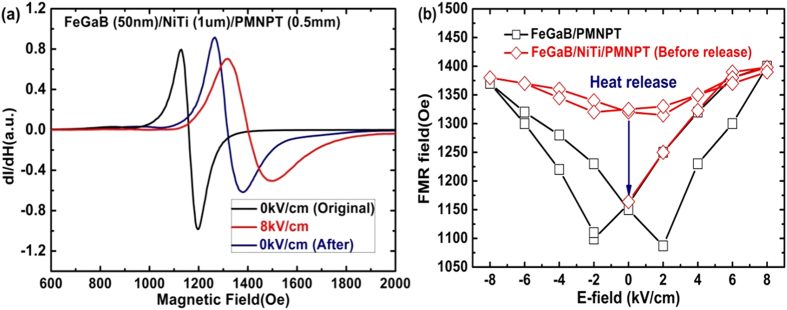
(**a**) Voltage control of FMR dependence in FeGaB/NiTi/PMNPT; (**b**) FMR field dependence of E-field before and after heat treatment.

The E-field dependent FMR spectra of the FeGaB/NiTi/PMN-PT heterostructure along easy axis under varied E-field are carried out with our ESR system, as shown in Fig. 2(a). The resonance occurs at 1158 Oe when there is no E-field applied across the sample. The FMR linewidth is approximately 30 Oe, which is wider than previously reported 16 Oe^62^. This may due to the increasing surface roughness, compared to Si surface, induced by the thick NiTi layer that is sandwiched between the PMN-PT single crystal and the FeGaB layer. When an E-field of 8 kV/cm is applied, strong ME interaction is observed in the FeGaB/NiTi/PMN-PT heterostructure, and the FMR up-shifted by 234 Oe to 1392 Oe. According to the Kittel’s equation^63^, the in-plane FMR can be expressed as:


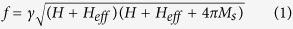


where 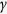 is the gyromagnetic ratio (~2.8 MHz/Oe), *H* is the external magnetic field, and 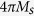 is the magnetization of FeGaB being 1.2 Tesla in this experiment. The E-field-induced uniaxial magnetic anisotropic field 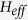 is


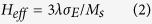


where λ and M_S_ is the saturation magnetostriction and magnetization of FeGaB, and 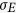 is E-field-induced biaxial stress. Moreover, the FMR sets at 1318 Oe after the electric bias is removed instead of at the original state 1158 Oe. This implies a partial non-volatile E-field control of FMR was obtained by introducing NiTi SMAs into multiferroic system. The reason could be that the memory effect of NiTi alloy in multiferroic heterostructure. NiTi strain driven by E-field did not release totally after removing E-field and it can sustain the FeGaB anisotropy without any external bias sources. Nevertheless, the FMR is not sustained at 1392 Oe, because the strain must be uniform at the NiTi and PMN-PT interface and the strain of the PMN-PT is decreased after the removal of the E-field. The NiTi strain is released to its original state after treated under a temperature of 200 °C. The NiTi phase transits from R-phase to a mixture R-phase and martensitic phase, resulting in a strain release between the two states. At this moment, the FMR field returns to ~1150 Oe, as shown in Fig. 2(b), the FMR field dependence of E-field. There is a significant difference between FeGaB/PMNPT multiferroic heterostructure with and without NiTi alloy. The ME coupling strength of FeGaB/NiTi/PMNPT (red) is much smaller than that of FeGaB/PMNPT (black) before heat treatment, see Fig. 2(b). It implies that the ME coupling is stored in the NiTi SMA and it can be released after heat treatment (blue arrow). This unique phenomenon will introduce novel functionality to voltage controllable ME devices, in which the voltage tunability can be controlled by heat or varied environments.

**Figure 3:**
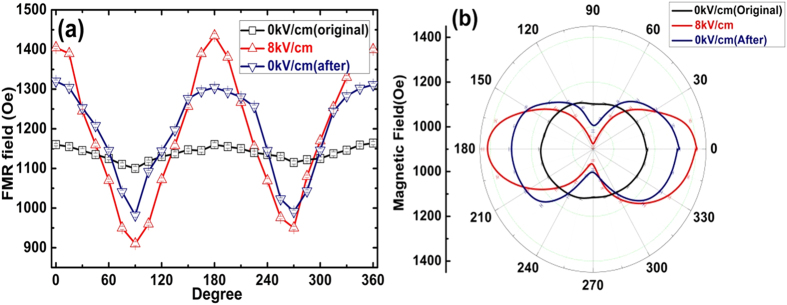
In-plane FMR angular dependence of E-field tuned FeGaB/NiTi/PMNPT (**a**) Cartesian coordinate (**b**) Polar coordinate.

In order to further study the magnetic anisotropy of the FeGaB/NiTi/PMN-PT sample, In-plane FMR angular dependence of E-field (0 and 8 kV/cm) is measured before and after thermal treatment, respectively. The sample is attached to a rotatable holder, and the measurement starts from the easy axis (E. A.) direction (defined as 0° and 360°) with a step size of 15°. The FMR field is 1160 Oe along the E. A (0°, 180°) and 1110 Oe along the hard axis (H. A.) direction (90° and 270°), see Fig. 3(a,b). The FMR field difference is due to the anisotropy induced by the *in situ* magnetic bias during deposition. The ME effect are observed in both E. A. and H. A. direction with opposite directions, which implies a uniaxial FMR field change, as shown in Fig. 3(a,b). After setting the E-field back to 0 kV/cm, the magnetic anisotropy reflected by the FMR field measurement is reduced, however, not fully recovered. By studying the magnetic anisotropy change, the memory effect of voltage tuning FMR field and partially non-volatile control of FMR field was obtained and confirmed in angular dependence study of FMR field shifts. It is worth to mention that out-of-plane FMR spectra is also very important that providing magnetic anisotropy change information. Nevertheless, we are focusing on studying memory effect in multiferroic FeGaB/NiTi/PZNPT in this paper. The In-plane FMR measurements provide sufficient information of strain/stress induce magnetic anisotropy change and the influence of NiTi layer.

**Figure 4:**
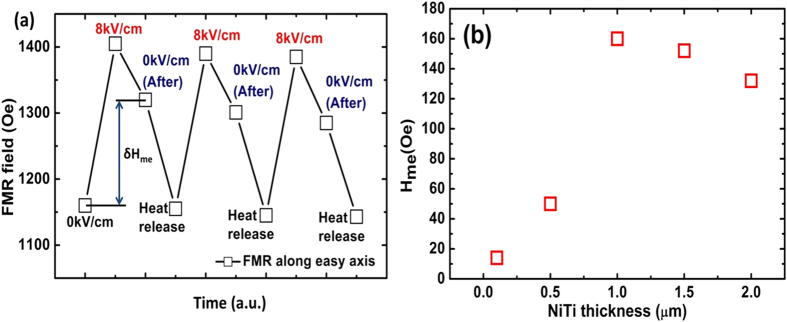
(**a**) FMR field tuning of FeGaB/NiTi/PMNPT in sequence; (**b**) memory effect strength (δHme) dependence of NiTi thickness.

Figure 4(a) illustrates the FMR field manipulated by E-field and heat impulse, forming a full cycles of magnetic anisotropy tuning in FeGaB/NiTi/PMNPT. At the beginning, the FMR field is switched from 1158 Oe to 1392 Oe by applying an 8 kV/cm E-field. Then, the FMR field is tuned back from 1392 Oe to 1318 Oe. Continually, the heat is released and the FMR of FeGaB/NiTi/PMNPT shifts back to the original state, forming an enclosed loop. A robust and repeatable non-volatile switching of effective magnetic field is achieved by dual E-field and heat impulse controlling. In Fig. 4(a), we define H_me_ as memory field represents the FMR field difference between FMR field at 8 kV/cm and FMR field at 0 kV/cm before heat release. A significant H_me_ dependence of NiTi thickness was obtained. At small NiTi thickness of 100 nm and 500 nm, the memory effects are small with H_me_<50 Oe; while the H_me_ is increasing up to 160 Oe at NiTi thickness of 1 μm. As NiTi thickness increases further, the H_me_ slightly decreases to ~130 Oe at NiTi thickness of 2 μm. A possible reason could be the memory effect is saturated at 1 μm, and further increasing of thickness could lead to high roughness that will degenerate the quality of films and also ME-memory effect. In general, it shows that with a NiTi SMA layer, extra FMR field state can be introduced into the multiferroic system, which opens a door for creating extra degree of freedom in multiferroic systems.

## Conclusions

In summary, we fabricated and studied the ME coupling behavior of a complex multiferroic heterostructure with FeGaB/NiTi/PMN-PT trilayer. The heterostructure produces a strong ME coupling anisotropy change of over 230 Oe under an external E-field of 8 kV/cm. Most importantly, by taking the advantage of the SMA NiTi, the heterostructure may store the ME coupling strength at room temperature and release it at a temperature of 200 °C. The voltage tunability of FeGaB magnetic anisotropy may be varied in different NiTi phase states through dual E-field and heat manipulation. As a result, a memorable ME coupling effect in created SMAs based multiferroic heterostructure is developed. The NiTi SMA based multiferroic heterostructure enables the smart multiferroic heterostructures works in varied environment, and it shows great potential in compact, fast tuning, and energy-efficient voltage tunable devices with multi-functionality.

## Methods

Multiferroic multilayers FeGaB/NiTi/PMN-PT were prepared by co-sputtering of Fe_80_Ga_20_ and B targets onto (011)-poled PMN-PT substrates (dimension 0.5 cm × 0.5 cm × 0.5 mm) coated with a layer of NiTi. All depositions were processed under a base pressure below 1 × 10^−7^ Torr at room temperature in magnetron sputtering system. The thickness of FeGaB and NiTi film are determined to be 50 nm and 1 um by fitting the X-ray reflectivity (XRR). Ferroelectric property of PMN-PT was measured by the radiant ferroelectric characterization system. The strain vs E-field curve was measured using a photonic sensor by sweeping the sinusoidal E-field. Moreover, the FMR spectra were measured using an X-band electron spin resonance (ESR) spectrometer in the field sweeping mode with a microwave frequency of 9.5 GHz and a power of 20 dBm. Sample heat treatment was done in an OVEN at 200 °C.

## Additional Information

**How to cite this article**: Zhou, Z. *et al.* The memory effect of magnetoelectric coupling in FeGaB/NiTi/PMN-PT multiferroic heterostructure. *Sci. Rep.* **6**, 20450; doi: 10.1038/srep20450 (2016).
